# Selective proteasome degradation of C‐terminally‐truncated human WFS1 in pancreatic beta cells

**DOI:** 10.1002/2211-5463.13674

**Published:** 2023-07-19

**Authors:** Hiraku Tokuma, Daisuke Sakano, Katsuya Tanabe, Yukio Tanizawa, Nobuaki Shiraki, Shoen Kume

**Affiliations:** ^1^ School of Life Science and Technology Tokyo Institute of Technology Yokohama Japan; ^2^ Division of Endocrinology, Metabolism, Haematological Science and Therapeutics Yamaguchi University Graduate School of Medicine Ube Japan

**Keywords:** beta cells, degradation, diabetes, endocrine, proteasome, WFS1

## Abstract

Wolfram syndrome is a monogenic disease mainly caused by mutations in the *WFS1* gene. Mutations in the *WFS1* gene give rise to diabetes. Here, we characterized mutant WFS1 proteins by studying the stability of full‐length wild‐type (WT) WFS1, a missense mutant P724L, and two C‐terminally truncated mutants, W837X and Y652X. We compared their stability by overexpressing them in MIN6 and HEK293T cells. The C‐terminally truncated mutants W837X and Y652X are degraded more rapidly than the missense P724L mutant or wild‐type WFS1 in MIN6 cells. In contrast, Y652X is more stable than WT or other mutant WFS1 proteins in HEK293T. In conclusion, we found that C‐terminally truncated WFS1 mutants are selectively degraded in a cell type‐specific manner.

## Graphical abstract

The *WFS1* gene is mutated in Wolfram syndrome, characterized by diabetes mellitus and optic atrophy. We compared the stability of full‐length WFS1 (wild‐type or a missense mutant) and C‐terminally truncated mutants by overexpressing them in a pancreatic beta cell or non‐pancreatic beta cell line. We found that the C‐terminally truncated mutants are selectively degraded in the pancreatic beta cell line.

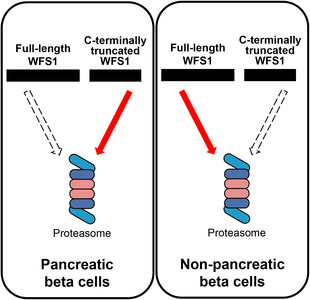

**Abbreviations**
DAPI: 4′,6‐diamidino‐2‐phenylindole
ER: endoplasmic reticulum
HEK: human embryonic kidney cells
HRP: horseradish peroxidase
hWFS1: human WFS1
MAMs: mitochondria‐associated ER membranes
MIN6: mouse insulinoma 6
ROI: region of interest
SD: standard deviation
TCID50: median tissue culture infectious dose
WFS1: Wolfram syndrome 1
WT: wild‐type

Wolfram Syndrome is an autosomal recessive disorder characterized by early‐onset insulin‐dependent diabetes mellitus optic atrophy and progressive neurodegeneration [1]. The *WFS1* gene is identified as the main locus mutated in the majority of the patients [2, 3]. Wolfram syndrome is characterized as a spectrum disorder. More than 200 different variations in the *WFS1* gene have been described in Wolfram syndrome patients [4, 5, 6, 7]. *WFS1* encodes a transmembrane protein, also called wolframin, which consists of 890 amino acids and is predicted to have 9 or 10 membrane‐spanning domains. The WFS1 protein is ubiquitously expressed but at higher levels in the brain, heart, lung, and pancreas [2, 3].

WFS1 is localized predominantly in the endoplasmic reticulum (ER) membrane [8] and appears to be enriched in mitochondria‐associated ER membranes (MAMs). Although the endogenous functions of WFS1 remain unclear, previous studies have pointed out the involvement of WFS1 in ER stress regulation and Ca^2+^ homeostasis [9, 10, 11, 12]. Recent studies revealed a role of WFS1 in regulating mitochondrial Ca^2+^ transfer through the MAMs [13, 14, 15, 16].

Mutations in the *WFS1* gene are reported to affect the WFS1 protein stability [17, 18, 19]. *Wfs1*‐knockout mice were developed [20, 21, 22], which exhibit enhanced ER stress, impaired insulin processing and regulation of insulin secretion and develop progressive glucose intolerance and increased pancreatic beta cell death.

Previous studies of genotype–phenotype correlations for WFS1 mutations in Japanese Wolfram syndrome patients revealed that WFS1 mutation could be subdivided into groups in correlation with clinical severity. Variants with nonsense mutations, frameshift mutations, and/or multiple amino acid insertion/deletions in both alleles show more severe symptoms than variants with missense mutations and/or single amino acid insertions in both alleles [7].

In the present study, we introduced one missense and two nonsense mutations in the *WFS1* cDNA to gain insights into the relationship between the severity of the mutations. We overexpressed the wild‐type (WT) and mutant WFS1 cDNAs and compared their stabilities in mouse insulinoma 6 (MIN6) cells, a mouse pancreatic β‐cell line, and HEK293T cells, a human embryonic kidney cell line.

## Materials and methods

### Cell lines

MIN6 cells were obtained from J. Miyazaki in Osaka University and HEK293T cells were from RIKEN BioResource Center (BRC) [23, 24].

### WFS1 mutant adenovirus vector construction

A cDNA fragment of human WFS1 WT open reading frame (nt. 1–2673; NP_005996.1 aa: 1–890) was cloned from human islet cDNA by PCR. Human WFS1 mutants were generated using inverse PCR and In‐Fusion HD (Takara Bio Inc., Shiga, Japan). Human WFS1 was used as the template for PCR. All primers for inverse PCR are listed in Table S1. The WFS1 mutant adenoviral vectors are registered at GenBank and listed in Table S2. Adenoviruses were produced as described [25]. Adenovirus titers were determined by Median Tissue Culture Infectious Dose (TCID50) [26].

### HaloTag pulse‐chase assay

**Fig. 1:**
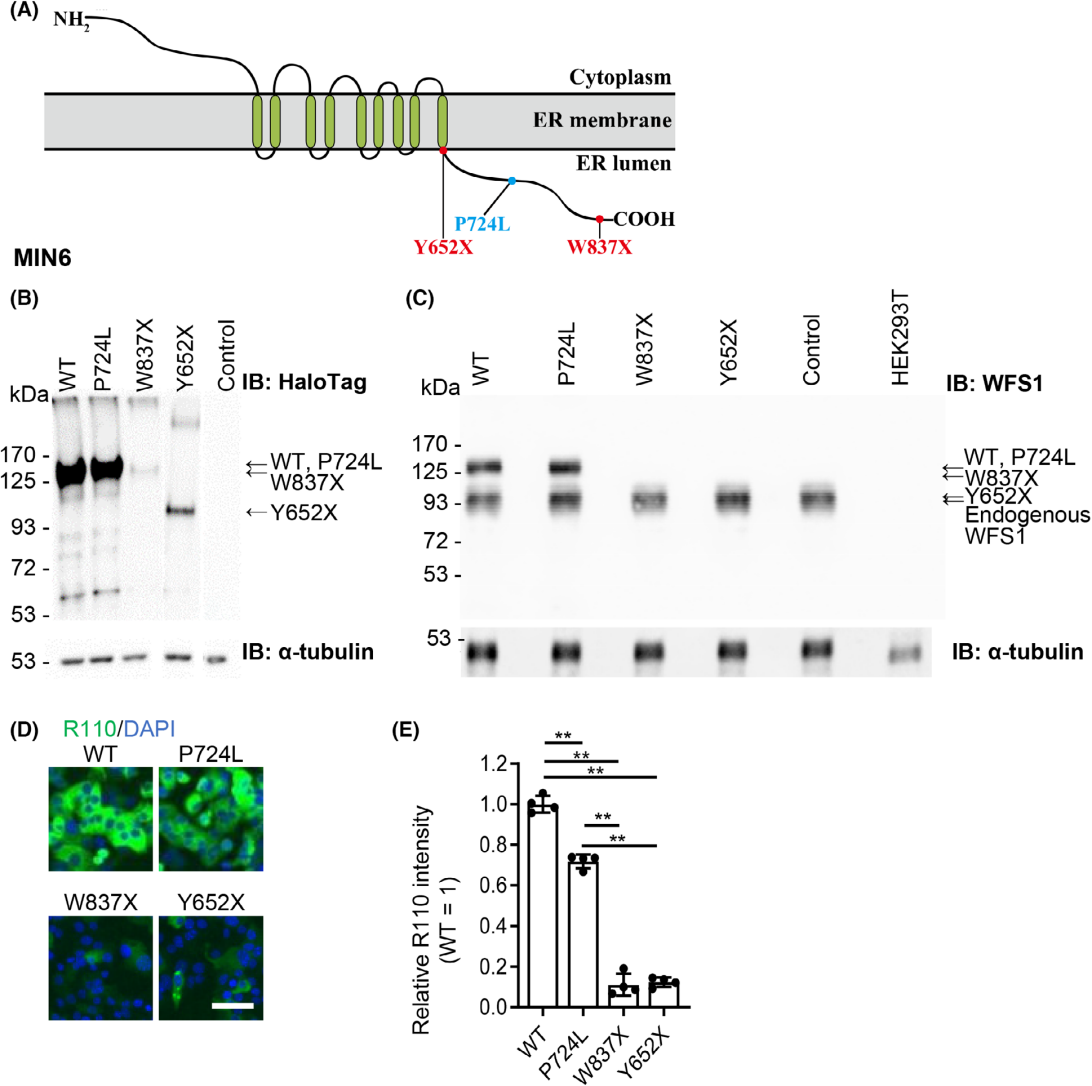
The C‐terminal deleted hWFS1 mutants are unstable in MIN6 cells. (A) A schematic drawing of the structure of the hWFS1 protein. Mutations indicated in red are nonsense or frameshift mutations. The Mutation indicated in blue is a missense mutation. (B, C) Whole‐cell lysates from MIN6 cells infected with adenovirus carrying HaloTag‐hWFS1 (WT, P724L, W837X, and Y652X), uninfected MIN6 cells (Control), and HEK293T cells were immunoblotted with anti‐HaloTag (B, upper panel), anti‐WFS1 (C, upper panel) or anti‐α‐tubulin antibody as internal controls (B, C lower panels). The steady‐state protein levels of C‐terminal deleted mutant hWFS1 (W837X, Y652X) were expressed at lower levels than HaloTag‐hWFS1 WT or P724L (B). The exogenous HaloTag‐hWFS1 WT or P724L were expressed at similar levels to the endogenous WFS1 (C). (D, E) Fluorescent images of the Halo ligand R110 labeled HaloTag‐hWFS1 WT or mutants in MIN6 cells (D). The relative intensities of fluorescence of R110 labeled HaloTag‐hWFS1 WT or mutant versus the WT WFS1 (=1) are shown (E). *N* = 4. (D) Scale bar = 50 μm. Data are presented as scattered plots with the means ± SD. Significant differences are shown as **P < 0.01, by one‐way ANOVA and Dunnett multiple comparisons test.

**Fig. 2:**
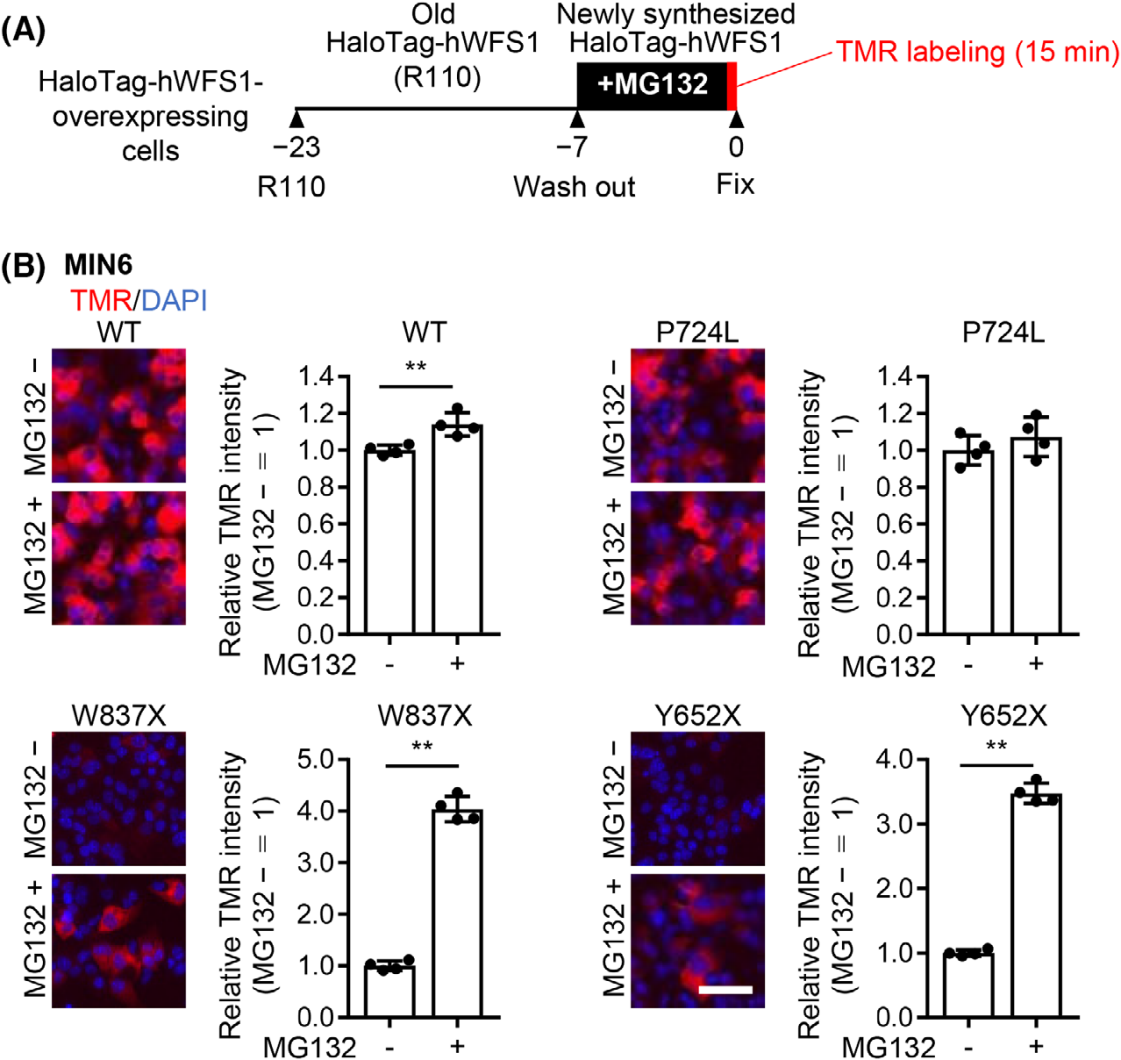
C‐terminally truncated hWFS1 proteins are degraded by the proteasome in MIN6 cells. (A) A schematic drawing of the pulse‐labeling with TMR using the HaloTag system. HaloTag‐hWFS1 overexpressing cells were incubated with R110 for 16 h to label the already produced ‘old’ HaloTag‐hWFS1. Then the cells were incubated for 7 h with or without MG132, then labeled the newly synthesized HaloTag‐hWFS1 with TMR. (B) Left panels: representative TMR fluorescence images of MIN6 cells. Right panels: relative average TMR intensities immediately after TMR labeling of the HaloTag‐hWFS1 proteins accumulated in MIN6 cells. Proteasome inhibitor MG132 promoted the accumulation of the WT WFS1 to 1.1‐fold and C‐terminally truncated HaloTag‐hWFS1 to 4.0‐fold (W837X) or 3.5‐fold (Y652X), versus without MG132. In contrast, MG132 did not promote the accumulation of the missense mutant (P724L). *N* = 4. Data are presented as scattered plots with the mean ± SD. Significant differences are shown as **P < 0. 01, by Student's *t*‐test. Scale bar = 50 μm.

**Fig. 3:**
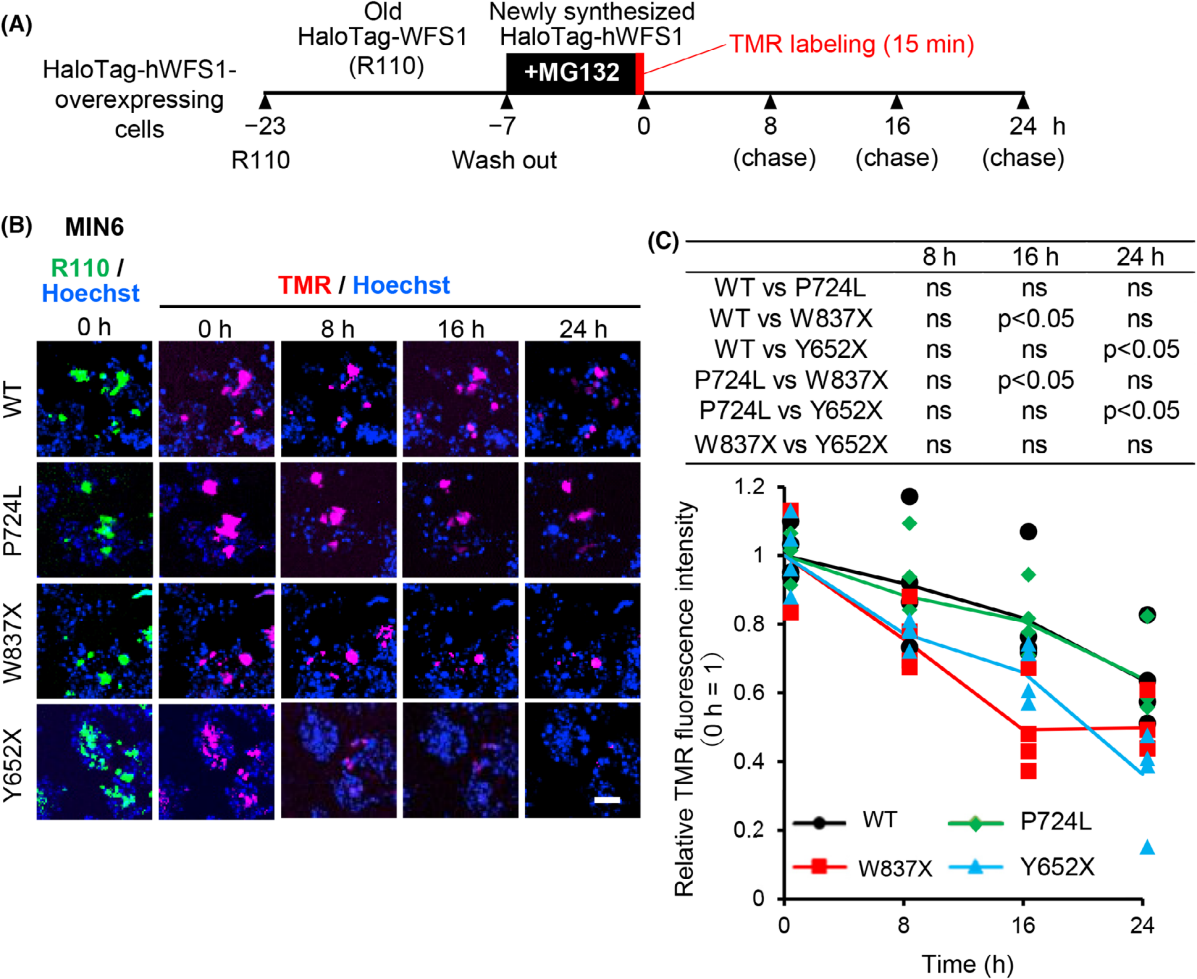
C‐terminally truncated W837X, and Y652X proteins are rapidly degraded in MIN6 cells. (A) A schematic drawing of the pulse‐chase experiment using the HaloTag system. HaloTag‐hWFS1 overexpressing cells incubated with R110 for 16 h (R110 labeled old hWFS1) were incubated with MG132 for 7 h, then labeled for 15 min with TMR (new hWFS1) and chased for the indicated times in the absence of MG132. (B) Representative time‐dependent TMR fluorescence images of WT or mutant WFS1 overexpressing MIN6 cells chased for 0, 8, 16, and 24 h. Scale bar = 50 μm. (C) R110 fluorescence of WT or mutant WFS1 protein expressing MIN6 cells, used for determining the ROIs. Time‐dependent average TMR fluorescence intensities of WT or mutant WFS1 in the pulse‐chase experiment in (B). Each point represents the average intensity of the ROI in each image. Cells were counter‐stained with Hoechst. The fluorescence intensities are expressed as fold‐change versus the WT or mutant WFS1 at 0 h, respectively. C‐terminally truncated WFS1 proteins were degraded more rapidly than full‐length WT and missense mutant P724L. *N* = 4. Data are presented as scattered plots with the mean ± SD. Significant differences were analyzed by Mann–Whitney's *U*‐test and are shown in a table. *P* < 0.05; ns, not significant.

**Fig. 4:**
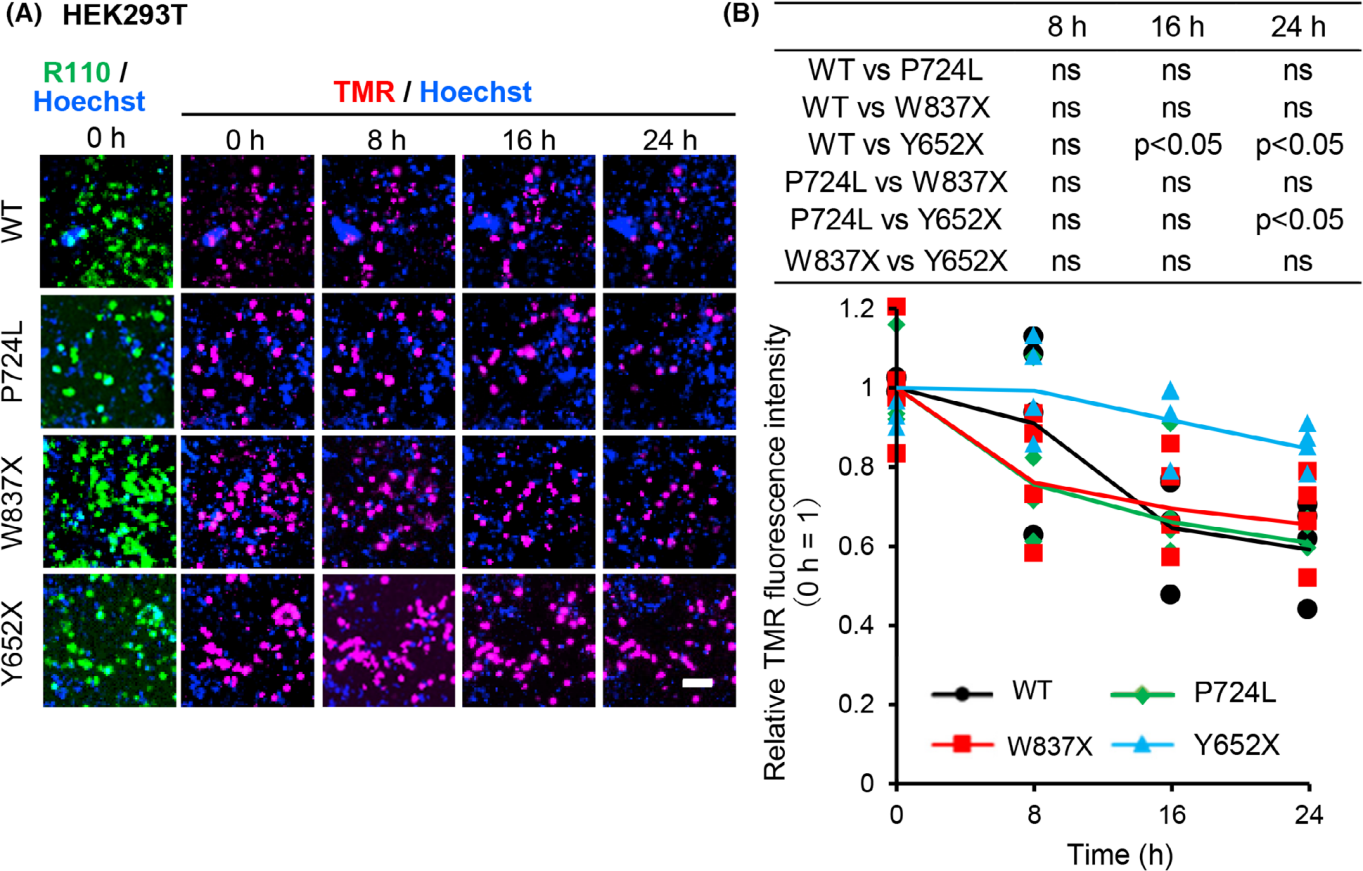
C‐terminally truncated Y652X mutant is more stable than WT, P724L or W837X hWFS1 in HEK293T cells. (A) HEK293T cells overexpressing HaloTag‐hWFS1 WT or mutants by adenovirus infection were treated similarly, with the old hWFS1 proteins labeled with R110, then incubated with MG132 for 7 h, then labeled for 15 with TMR to label new hWFS1 and chased for the indicated times in the absence of MG132. Representative time‐dependent TMR fluorescence images of HaloTag‐hWFS1 WT or mutant overexpressing HEK293T cells chased for 0, 8, 16, and 24 h. Cells were counter‐stained with Hoechst. Scale bar = 50 μm. (B) R110 fluorescence of WT or mutant WFS1 protein expressing HEK293T cells, used for determining the ROIs. Time‐dependent average TMR fluorescence intensities of HaloTag‐hWFS1 WT or mutants in the pulse‐chase experiment in HEK293T cells. Each point represents the average intensity of each image. The fluorescence intensities are expressed as fold‐change versus the WT or mutant WFS1 at 0 h, respectively. *N* = 4. Data are presented as scattered plots with the mean ± SD. Significant differences were analyzed by Mann–Whitney's *U*‐test and are shown in a table. *P* < 0.05; ns, not significant.

MIN6, HEK293T cells were cultured in Dulbecco's modified Eagle's medium supplied with 25 mm glucose (Thermo Fisher Scientific Inc., Waltham, MA, USA), 10% fetal bovine serum, 100 μm non‐essential amino acids, 2 mm l‐glutamine, 50 units·mL^−1^ penicillin, 50 μg·mL^−1^ streptomycin, and 100 μm 2‐mercaptoethanol, at 37 °C, 5% CO_2_. Cells were dissociated with 0.05% Trypsin–EDTA (Thermo Fisher Scientific) and seeded on a 96‐well plate coated with Easy iMatrix‐511 (Matrixome Inc., Osaka, Japan) at a density of 50 000 cells per well (MIN6 cells) or 10 000 cells (HEK293T cells). MIN6 cells were infected with adenovirus carrying *HaloTag‐hWFS1* or *hWFS1* mutants to examine the steady state of the protein expression in Fig. 1 and in the pulse‐labeling experiment in Fig. 2. The multiplicity of infection was 3.3. In the pulse‐chase experiment, *HaloTag‐hWFS1* or mutants were inserted into the pCAGGS vector [27] and transfected using Lipofectamine 3000 (Thermo Fisher Scientific) in Figs 3 (MIN6) and 4 (HEK293T). *HaloTag‐hWFS1* overexpressing vectors are registered at GenBank and listed in Table S2. Labeling using R110 or TMR was done following the manufacturer's protocol. After infection or transfection, cells were cultured for 32 h, then treated with HaloTag R110Direct™ ligand (R110; Promega, Madison, WI, USA) for 16 h to bind to the already produced ‘old’ HaloTag‐hWFS1. After washing with medium to remove the R110, cells were cultured in the presence of a proteasome inhibitor, MG132 (10 μm, in the pulse‐chase experiment), or control DMSO (in the pulse labeling). Then, cells were treated with HaloTag TMR ligand (TMR; Promega) for 15 min (±MG132) to label newly synthesized HaloTag‐hWFS1. After pulse labeling with TMR, the steady‐state expression levels of the WFS1 proteins were examined after fixing the cells in 4% paraformaldehyde in PBS and counter‐stained with DAPI (F. Hoffmann‐La Roche Ltd., Basel, Switzerland) (Fig. 2). R110 or TMR fluorescence that labeled the HaloTag‐hWFS1 in the cells was analyzed with ImageXpress Micro Confocal High‐Content Imaging System and MetaXpress (Molecular Devices, San Jose, CA, USA). To chase the TMR‐labeled HaloTag WT or mutant hWFS1 proteins, cells were counter‐stained with Hoechst 33342 (Bio‐Rad Laboratories Inc., Richmond, CA, USA) and cultured for 8, 16, or 24 h in the absence of MG132 (Figs 3 and 4). The areas of cells expressing the WFS1 proteins were determined as region of interest (ROI) based on the 0 h image labeled with R110. The averaged TMR fluorescence intensity values were obtained for each ROI at 0, 8, 16, and 24 h after TMR treatment. All calculations were performed using the image j fiji program [28] for images taken by imagexpress.

### Western blot analysis

Cells were lysed with 2 × Laemmli buffer [0.1 mm Tris–HCl (pH 6.8), 4% SDS, 20% Glycerol, 1.7 m 2‐mercaptoethanol] or with loading buffer [62.5 mm Tris–HCl (pH 6.8), 2% SDS, 10% glycerol and 41.6 mm dithiothreitol] and sonicated for 20 s, and incubated overnight at 55 °C. Samples were separated using an 8% acrylamide gel and transferred onto a polyvinylidene difluoride membrane (Merck Millipore Ltd, Billerica, MA, USA) and incubated overnight at 4 °C with mouse anti‐HaloTag (1 : 1000, Cat: G9211; Promega), rabbit anti‐WFS1 (1 : 1000) [29], or mouse anti‐α‐tubulin (1 : 2000, Cat: T6074; Sigma, Billerica, MA, USA). After washing with PBST (PBS containing 0.1% Tween20), the membrane was probed with a horseradish peroxidase‐conjugated antibody. The membrane was scanned using FUSION‐SOLO.4S.WL (M&S Instruments Inc., Tokyo, Japan) after reacting with Luminata Forte Western HRP Substrate (Merck Millipore).

### Statistics

Data are presented as mean ± SD (standard deviation) with individual data. Data were analyzed by one‐way ANOVA and Dunnett multiple comparisons test (Fig. 1D), Student's *t*‐test (Fig. 2B), or Mann–Whitney's *U*‐test (Figs 3C and 4B). Significant differences are shown as **P < 0.01 or *P* < 0.05 in the tables. All data were obtained from more than three independent experiments except those specifically indicated.

## Results

### The hWFS1 C‐terminally truncated mutants are unstable in MIN6 cells

We investigated one missense and two C‐terminally truncated human *WFS1* (*hWFS1*) mutations. The missense mutation corresponds to the luminal C‐terminal (P724L), and the C‐terminal truncation mutations Y652X and W837X lead to the truncation of a large portion of the C‐terminus end (Y652X) or truncation of the last 54 amino acid residues (W837X) (Fig. 1A). The patients with the P724L mutation were previously described as late onsets of diabetes mellitus, compared to those with the W837X and Y652X mutations, who developed early onset diabetes [7].

To assess the steady‐state expression of the hWFS1 mutants, we generated adenoviral vectors carrying human WT or mutant hWFS1 tagged with HaloTag [30] at their N‐terminal ends. We infected the MIN6 cell with the above adenoviral vectors and detected by western blot analysis using an anti‐HaloTag antibody or anti‐WFS1 antibody. The exogenous HaloTag WFS1 WT or the missense hWFS1 mutant P724L gave strong signals at approximately 134 kDa. In contrast, the C‐terminally truncated hWFS1 mutants Y652X and W837X expressed in MIN6 cells gave weak signals at 127.7 kDa (W837X) or 106.8 kDa (Y652X) (Fig. 1B). In MIN6 cells, the exogenous HaloTag WFS1 WT and P724L were expressed at almost similar levels with those of the endogenous WFS1, detected by an anti‐WFS1 antibody (Fig. 1C). In W837X and Y652 overexpressed MIN6 cells, the endogenous WFS1 bands were also detected, although the overexpressed HaloTag‐W837X or Y652X were undetectable (Fig. 1C).

We also used a synthetic Halo ligand R110 that binds to the HaloTag‐hWFS1 protein. The R110 fluorescence visualizes the expression of the WT or mutant WFS1 (Fig. 1D,E). Representative images are shown in Fig. 1D. Average fluorescence intensities in the ROIs are shown in scattered plots (Fig. 1E). The results revealed that the P724L missense mutation was expressed at approximately 0.7‐fold of that of the WT. The C‐terminally truncated mutants W837X and Y652X were expressed at 0.1‐fold of the WT (Fig. 1D,E). The results suggest an instability of the C‐terminally truncated hWFS1 mutations expressed in MIN6 cells.

### C‐terminally truncated hWFS1 proteins are rapidly degraded by the proteasome in MIN6 cells

It is reported that WFS1 mutants are degraded by the ubiquitin‐proteasome system [17, 18]. We investigate whether the instability of the WFS1 mutant proteins expressed in MIN6 cells is through proteasome degradation. After labeling the already produced ‘old’ HaloTag‐hWFS1 with R110, the HaloTag‐hWFS1 overexpressing MIN6 cells were incubated for 7 h with or without proteasome inhibitor MG132, then labeled the newly synthesized HaloTag‐hWFS1 protein with TMR ligand (Fig. 2A, pulse labeling with TMR). The results showed that TMR‐labeled WT and P724L mutant hWFS1 protein accumulated even without MG132. MG132 treatment increased the accumulation of WT or P724L hWFS1 to about 1.1‐fold versus the untreated group (Fig. 2B, right). In contrast, the C‐terminally truncated hWFS1 mutants were detected at low levels in the absence of MG132. In the presence of MG132, the C‐terminally truncated mutants W837X or Y652X accumulated up to approximately 4.0‐fold (W837X) or 3.5‐fold (Y652X) in MIN6, compared to without MG132 (Fig. 2B).

Adding MG132 rescued the degradation of the C‐terminally truncated mutants, indicating the proteasome‐mediated mechanism is involved. To analyze this in detail, we performed another pulse‐chase labeling experiment (Fig. 3A). The R110‐labeled HaloTag‐hWFS1 expressing MIN6 cells (overlapped with the TMR‐labeled cells) (0 h) were used to determine the ROIs. TMR‐labeled HaloTag‐hWFS1 proteins were chased for 8, 16, and 24 h. As a result, WT and P724L proteins were reduced to about 0.65‐fold after incubating without MG132 for 24 h. In contrast, C‐terminally truncated mutants W837X and Y652X were rapidly degraded to about 0.40‐ to 0.50‐fold at 16 h (W837X) or 24 h (Y624X) in MIN6 cells (Fig. 3B,C).

The results indicate a proteasome‐mediated rapid degradation of C‐terminally truncated mutants W837X and Y652X in MIN6 cells.

### C‐terminally truncated mutant Y652X is more stable in HEK293T cells compared to WT or other WFS1 mutants

We then examined the stability of WT and mutants in HEK293T cells. We performed similar pulse‐chase experiments in HEK293T cells. HEK293T cells expressed WFS1 endogenously at a much lower level than MIN6 cells (Fig. S1). The newly synthesized HaloTag‐WT or mutant hWFS1 proteins expressed in the HEK293T cells were labeled with TMR (pulse, 0 h) and were chased for 8, 16, and 24 h in the absence of MG132.

Without MG132, the WT and the missense P724L mutant were degraded to approximately 0.60‐fold (Fig. 4A,B), and the C‐terminally truncated mutant W837X was about 0.65‐fold at 24 h. In contrast, the C‐terminally truncated mutant Y652X was about 0.85‐fold at 24 h. The results indicate that the C‐terminally truncated Y652X mutant is the most stable form in HEK293T cells compared to the WT or the P724L mutant (Fig. 4A,B).

## Discussions

We compared the stability of WT and mutant WFS1 protein in MIN6 and HEK293T cells. The C‐terminus truncated mutants W652X and W837X are degraded by the proteasome pathway in MIN6 cells. In contrast, the C‐terminally truncated mutant W652X is more stable than WT or other WFS1 mutants in HEK293T cells. MG132 treatment did not restore the C‐terminally truncated mutants to accumulate to the WT level, suggesting the involvement of other non‐proteasomal mechanisms. The result suggests that protein stability depends on the tissue or cell environment, which agrees that protein stability varies among tissues [31].

Our results agree with a previous report that the C‐terminally truncated mutants exhibited higher stabilities than the WT WFS1 in HEK293T cells [18]. However, the exact mutants used between ours and the previous studies differ. Notably, WT and mutant WFS1 seem to accumulate at similar levels at a steady state in HEK293T cells [18], which agrees with our present results. In MIN6 cells, the steady‐state accumulations of C‐terminus truncated mutants W837X and T652X were approximately 0.1‐fold of the WT WFS1 protein, which was much lower compared to HEK293T cells. The results indicate that WFS1 WT and mutants are more stable in HEK293T than in MIN6 cells. A selective degrading mechanism of C‐truncated WFS1 mutants seems to exist in beta cells. In MIN6 cells, the endogenous WFS1 was not degraded by overexpressing W837X or Y652X mutants, thereby confirming that the degradation system was not activated. The discrepancies between the stability of the WT and mutant WFS1 in MIN6 and HEK293T cells might be due to the different expression levels in regulating proteins involved in the degradation processes in a cell type‐specific manner, which remains to be elucidated.

It is reported that the HECT‐type ligase ubiquitin ligase (E3) Smad ubiquitination regulatory factor 1 (Smurf1) binds to the C‐terminus of WFS1 and degrades WFS1 thus contributing to the instability in HEK293T cells [18]. However, the C‐terminally truncated WFS1 mutants are unstable in MIN6 cells, suggesting a selective degrading mechanism of C‐terminally truncated WFS1 mutants in beta cells. Expression of other members of the E3 ubiquitin ligase or other targeted protein degrading mechanisms, such as ERAD or SUMOylation, are reported in the pancreatic beta cells and might take part in regulating protein stability. However, we did not find any motif existing in the WFS1 protein [10, 32, 33, 34, 35, 36, 37].

Understanding the molecular mechanism regulating WFS1 stability might provide a potential therapeutic target for Wolfram syndrome.

## Conflict of interest

The authors declare no conflict of interest.

## Author contributions

HT performed the experiments, collected, analyzed, and discussed the data. DS discussed the data and provided conceptual input and technical advice. KT, YT, and NS discussed the data and gave technical advice. SK provided conceptual input, discussion, writing, and revision of the manuscript; approved the final version of the manuscript; and obtained funding.

### Peer review

The peer review history for this article is available at https://www.webofscience.com/api/gateway/wos/peer‐review/10.1002/2211‐5463.13674.

## Supporting information


**Fig. S1.** HEK293T cells express WFS1 endogenously at a much lower level compared to MIN6 cells.
**Table S1.** Primers used for generating WFS1 mutants.
**Table S2.** The WFS1 mutant vectors.

## Data Availability

The data supporting this study's findings are openly available in GenBank at https://www.ncbi.nlm.nih.gov/genbank/, and the accession numbers are listed in Table S2.
