# Supplementary material for: Selective proteasome degradation of C‐terminally‐truncated human WFS1 in pancreatic beta cells
Source: FEBS Open Bio. 2023 Jul 19;13(8):1405–14. doi: 10.1002/2211-5463.13674 (PMC10392043; doi:10.1002/2211-5463.13674)
Supplement: Supplementary file 1 — Fig. S1. HEK293T cells express WFS1 endogenously at a much lower level compared to MIN6 cells. Table S1. Primers used for generating WFS1 mutants. Table S2. The WFS1 mutant vectors. [file FEB4-13-1405-s001.pdf]

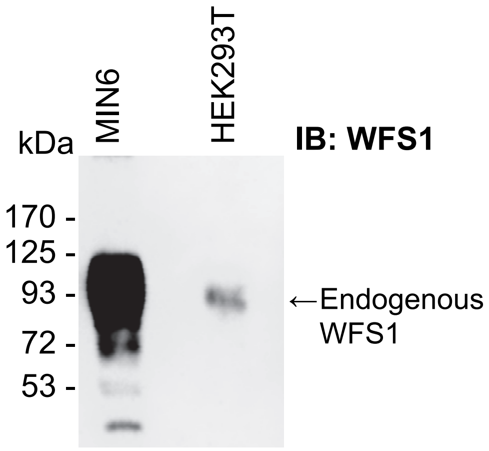

Supplementary Figure S１. HEK293T cells express WFS1 endogenously at a much lower level compared to MIN6 cells.
Endogenous WFS1 expressions in MIN6 or HEK293T cells were detected by western blot analysis using an anti-WFS1 antibody.

Supplementary Table S1. Primers used for generating WFS1 mutants

WFS1 mutant	Forward primer	Reverse primer
Wild-Type	ATGGACTCCAACACTGCTCCG	GGAGCTCCAATTCGCAGGCCTCATGGCAACATG
Y652X	TATGTGTAACGCTCAGAGGGCATG	TGAGCGTTACACATAGAACCAGCAG
P724L	ATGCTCCTGTTCTTCATCGGC	GAAGAACAGGAGCATGTTGATGG
W837X	GCAAGTGACCTGTCTTCGAGCTCAAG	AGACAGGTCACTTGCTGCCCAG

Supplementary Table S2. The WFS1 mutant vectors

Name	Description	Accession or Catalog Number
pENTR1A-HaloTag-hWFS1 (WT)	Entry vector carrying human WFS1 wild-type fused with HaloTag at N-terminus	Accession No. OQ102498
pAd/CMV/V5-DEST-HaloTag-hWFS1 (WT)	Adenoviral vector for overexpression of HaloTag-hWFS1 (WT)	Accession No. OQ200484
pCAG-HaloTag-hWFS1 (WT)	Expression vector of HaloTag-hWFS1 (WT) under the control of CAG promoter	Accession No. OQ362366
pENTR1A-HaloTag-hWFS1 (Y652X)	Entry vector carrying human WFS1 Y652X fused with HaloTag at N-terminus	Accession No. OQ200483
pAd/CMV/V5-DEST-HaloTag-hWFS1 (Y652X)	Adenoviral vector for overexpression of HaloTag-hWFS1 (Y652X)	Accession No. OQ200486
pCAG-HaloTag-hWFS1 (Y652X)	Expression vector of HaloTag-hWFS1 (Y652X) under the control of CAG promoter	Accession No. OQ362368
pENTR1A-HaloTag-hWFS1 (P724L)	Entry vector carrying human WFS1 P724L fused with HaloTag at N-terminus	Accession No. OQ238869
pAd/CMV/V5-DEST-HaloTag-hWFS1 (P724L)	Adenoviral vector for overexpression of HaloTag-hWFS1 (P724L)	Accession No. OQ362365
pCAG-HaloTag-hWFS1 (P724L)	Expression vector of HaloTag-hWFS1 (P724L) under the control of CAG promoter	Accession No. OQ362369
pENTR1A-HaloTag-hWFS1 (W837X)	Entry vector carrying human WFS1 W837X fused with HaloTag at N-terminus	Accession No. OQ200482
pAd/CMV/V5-DEST-HaloTag-hWFS1 (W837X)	Adenoviral vector for overexpression of HaloTag-hWFS1 (W837X)	Accession No. OQ200485
pCAG-HaloTag-hWFS1 (W837X)	Expression vector of HaloTag-hWFS1 (W837X) under the control of CAG promoter	Accession No. OQ362367
pCAGGS	Control vector in HEK293T	RIKEN BRC, Cat No. RDB08938
